# Ecosystem Services at the Farm Level—Overview, Synergies, Trade‐Offs, and Stakeholder Analysis

**DOI:** 10.1002/gch2.202200225

**Published:** 2023-04-27

**Authors:** Jonathan Morizet‐Davis, Nirvana A. Marting Vidaurre, Evelyn Reinmuth, Esmaeil Rezaei‐Chiyaneh, Valentin Schlecht, Susanne Schmidt, Kripal Singh, Ricardo Vargas‐Carpintero, Moritz Wagner, Moritz von Cossel

**Affiliations:** ^1^ Biobased Resources in the Bioeconomy (340b) Institute of Crop Science University of Hohenheim 70599 Stuttgart Germany; ^2^ Department of Plant Production and Genetics at Urmia University Urmia Iran; ^3^ School of Agriculture and Food Sciences University of Queensland The University of Queensland Brisbane 4072 QLD Australia; ^4^ Department of Biological Sciences and Biotechnology Andong National University Andong 36729 Republic of Korea; ^5^ Department of Applied Ecology Hochschule Geisenheim University 65366 Geisenheim Germany

**Keywords:** agricultural production, digital farming, ecosystem integrity, resilience, sustainable production and consumption

## Abstract

The current geological epoch is characterized by anthropogenic activity that greatly impacts on natural ecosystems and their integrity. The complex networks of ecosystem services (ESs) are often ignored because the provision of natural resources, such as food and industrial crops, is mistakenly viewed as an independent process separate from ecosystems and ignoring the impacts on ecosystems. Recently, research has intensified on how to evaluate and manage ES to minimize environmental impacts, but it remains unclear how to balance anthropogenic activity and ecosystem integrity. This paper reviews the main ESs at farm level including provisioning, regulating, habitat, and cultural services. For these ESs, synergies are outlined and evaluated along with the respective practices (e.g., cover‐ and intercropping) and ES suppliers (e.g., pollinators and biocontrol agents). Further, several farm‐level ES trade‐offs are discussed along with a proposal for their evaluation. Finally, a framework for stakeholder approaches specific to farm‐level ES is put forward, along with an outlook on how existing precision agriculture technologies can be adapted for improved assessment of ES bundles. This is believed to provide a useful framework for both decision makers and stakeholders to facilitate the development of more sustainable and resilient farming systems.

## Graphical abstract

In agricultural production, there are many ecosystem services other than biomass provision that are strongly influenced by farming practices. In order to provide more credit to farmers for more sustainable management, stakeholders need to be identified and additional farm‐level data generated. This review critically discusses how this can be done for more sustainable and resilient agriculture.

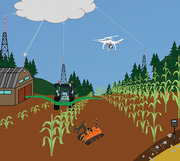

## 1 Introduction

The current geological epoch is characterized by anthropogenic activity that dominates natural ecosystems^[^ ^1^, ^2^, ^3^, ^4^ ^]^ and greatly impacts the integrity of the natural environment.^[^ ^5^, ^6^ ^]^ This integrity is tied together by complex networks of ecosystem services (ES).^[^ ^7^ ^]^ Over the past decade, research has intensified on how to evaluate and manage these ES to minimize environmental impacts of business and everyday life.^[^ ^8^ ^]^ Concepts such as civic ecology,^[^ ^9^ ^]^ sustainable development,^[^ ^10^, ^11^, ^12^ ^]^ and the bioeconomy^[^ ^13^, ^14^ ^]^ are being rapidly operationalized and often integrate ecological practices into their implementation strategies, by way of how we interact with nature. In this regard, philosopher and scientist Aldo Leopold wrote in the mid‐20th century: “*A thing is right when it tends to preserve the integrity, stability, and beauty of the biotic community. It is wrong when it tends otherwise*.”^[^ ^15^ ^]^ Similarly, the paradigm of civic ecology^[^ ^9^ ^]^ is founded in subjective philosophy, which aims to expand our awareness of the natural world and the systems in place.

One of the fundamental practices in civic ecology is systems thinking, with the idea that individuals should form a relation to nature, like they do to a human community. Further, systems thinking is frequently used in science to understand how individuals interact with the system. For instance, an ecosystem shares this attribute, where a dynamically evolving system is influenced by interacting, individual elements including air, water, soil, microbes, plants, and animals. Unfortunately, this notion is often ignored, because the provisioning of natural resources is seen as an independent act separate to the ecosystems, thus disregarding the impact of anthropogenic changes in ecosystems.^[^ ^2^, ^16^ ^]^ During the Anthropocene, humans can benefit from system thinking by finding more harmonious ways to interact with nature,^[^ ^17^ ^]^ for example by moving toward social‐ecologically more sustainable cropping systems.^[^ ^18^, ^19^ ^]^

So how is it possible to balance anthropogenic activity while maintaining the ecosystem's integrity of the farm and the surrounding land? This is a complex challenge, because it requires considering both the time‐ and scale‐dependent interactions between ES and a shared consensus among communities, countries, and nations at large.^[^ ^20^, ^21^, ^22^ ^]^ Moreover, the fundamental question of the value of nature is at the core of this challenge.^[^ ^23^, ^24^ ^]^ One economic‐based proposition is to monetize the ecosystem and its services,^[^ ^7^, ^25^ ^]^ despite its limitation to capture the plural, complex and subjective characteristics of “value of nature” and its rather instrumental and monistic approach.^[^ ^24^, ^26^ ^]^ The ecosystem and its services might be monetized in a way which applies value onto the services that nature has to offer, producing shared monetary incentives among stakeholders.^[^ ^27^, ^28^, ^29^, ^30^ ^]^ Across the USA, the estimated value of ES is within US$ 33 trillion but the real value of these services is potentially far greater and yet to be uncovered.^[^ ^7^ ^]^ A conventional way of ES monetization is through the provisioning of agricultural goods.^[^ ^30^ ^]^ In fact, agriculture, i.e., the generation and use of ES in agricultural production, is one of the oldest known ecological practices,^[^ ^31^ ^]^ and agriculture has greatly impacted life on the planet.^[^ ^6^, ^32^ ^]^ Research suggests that ecofriendly farming systems have the potential to provide enormous benefit for both humans and the natural environment,^[^ ^33^, ^34^ ^]^ as a well‐managed ecosystem can help ensure that its services are regenerative for indefinite use.^[^ ^31^, ^35^ ^]^ However, this is a major challenge, as Braat and De Groot (2012) stated that “*Ecosystems produce multiple services and these interact in complex ways, different services being interlinked, both negatively and positively*.” Thus, for a balanced agroecosystem management, an ES approach at the farm level is preferable to a plot or field level due to the numerous dependencies and interactions that cross field boundaries.^[^ ^36^, ^37^ ^]^

Therefore, this paper examines the benefit of natural ecosystems in farming by investigating the values, types, and uses of ES at the farm level. This article has three main objectives, the first is to identify the ES of a farm and how they may be incorporated into agricultural production to benefit the farm (Section 3.1). The second objective is to discuss the synergies and trade‐offs of various services, as ecosystems typically adapt to their surroundings so that alterations must be carefully analyzed to avoid unintended negative impacts (Sections 3.2 and 3.3).^[^ ^35^ ^]^ The third objective is to help create transparency among stakeholders and to provide an outlook on the role of digital farming for ecosystem service assessment (Sections 3.4 and 3.5).

## 2 Experimental Section

To address the research objectives, a literature review was conducted. Due to the diversity of disciplines and document types related to ecosystem services, a broad screening of relevant literature from various databases such as Scopus (Elsevier B.V., Amsterdam, The Netherlands), and Google Scholar (Google LLC, California, United States of America) was conducted. A variety of key terms were used for this screening, but the number of documents found was not documented. The bibliographies of the documents found were also considered.

## 3 Results and Discussion

### 3.1 Ecosystem Services at Farm Level

**Figure 1:**
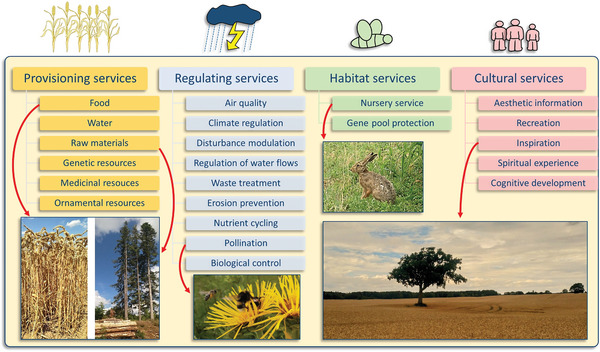
Overview of the four main ecosystem service categories and illustrating key ecosystem services at farm level that are vital for sustainable farming systems (categories adapted from De Groot et al.^[^ ^25^ ^]^).

The web of ES is intangible in the sense that we cannot see it occurring in its complexity (**Figure** **1**). However, research and conventional knowledge have contributed to a better understanding of how ecosystems work at different levels, and how to benefit ES by regulating the systems. It is possible to generate synergistic relationships among naturally occurring species of plants, animals, and microbes while adjusting the system's interactions.^[^ ^3^ ^]^ These synergies are known as symbiotic interactions, and they occur when two or more species interact with one another for mutual benefit.^[^ ^38^ ^]^ Soils are important components of ES as they support microorganisms and plants as essential drivers of biogeochemical cycles.^[^ ^39^ ^]^ Soil, microbes and plants have the power to influence nutrient composition, water retention, carbon capture and storage, and atmospheric oxygen levels.^[^ ^35^ ^]^ For example, microbes in the upper soil produce a significant amount of the Earth's oxygen.^[^ ^40^ ^]^ Climate research suggests that by applying the systems concept to agricultural production, ecosystem management has the capacity to transform ecosystems at the biome scale while regulating the Earth's atmosphere at the macro scale. As a result, effective management of agricultural production is crucial in addressing climate change, which has far‐reaching implications beyond the farm level – even with regard to soil microbes and plants, which are commonly more associated with the field level.^[^ ^41^ ^]^

The farm‐level approach demonstrates the importance of adequate ES management in mitigating climate change while ensuring food supply for humanity. However, to correctly administer these services, one must first become acquainted with their main types. There are several categories of these ES main types in the literature, with de Groot et al. (2012) being one of the most widely used: provisioning services, regulating services, habitat services (supporting services) and cultural services (Figure 1).^[^ ^25^ ^]^ This ES classification is used in the following.

#### 3.1.1 Provisioning Services

**Figure 2:**
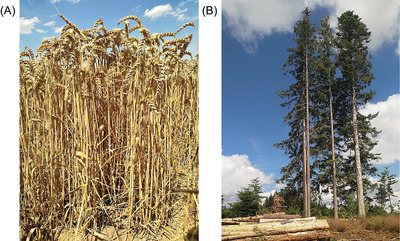
Providing ecosystem services include, but are not limited to, providing biomass for A) food sources such as winter wheat (*Triticum aestivum* L.) and B) fuels and biobased materials from wood.

Provisioning services offer the greatest tangible benefits,^[^ ^2^ ^]^ whose product is renewable as long as it is grown or cultivated. Harvesting organic materials for use in food (**Figure** **2**A), feed, fiber, chemicals, or fuels (Figure 2B) are examples of provisioning ES activity.^[^ ^42^ ^]^

In general, these advantages have a monetary value and can be exchanged as a commodity.^[^ ^30^ ^]^ Among these services, food is anticipated to be the most common extracted resource. Food can be obtained in different ways, including agricultural techniques, fisheries, aquaculture, hunting, wild harvesting, and livestock farming. Biomass can also be grown in agriculture as a feedstock or converted into bioenergy.^[^ ^43^ ^]^ Fibers derived from biomass, such as cotton, can be used in the production of clothing. Furthermore, biomass from provisioning services can be used as animal feed or fertilizer for other crops. It can also be used to cultivate and refine high‐quality chemicals and pharmaceuticals. Last but not least, agroecosystems provide water or at least influence the quality of the water that flows through the soil. Overall, the mean total monetary value of the provisioning services strongly depends on the location and the type of biome such as grassland (Int.$1337 per hectare and year) and cultivated land (Int.$2120 per hectare and year), which explains high standard deviations.^[^ ^44^ ^]^ In the case of mixed farming, for example, fish culture in wet rice plantations, even much higher revenues can be generated from provisioning services ranging from $1469^[^ ^45^ ^]^ to $11192 per hectare per year.^[^ ^46^ ^]^

#### 3.1.2 Regulating Services

Regulating services can function as a control variable for the agri‐ecosystem. The mean total monetary value of regulating services is estimated to account for about Int.$3453 per hectare and year for grassland and Int.$8096 per hectare and year for cultivated land.^[^ ^44^ ^]^

**Figure 3:**
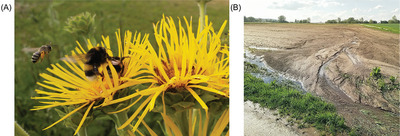
If managed well, farmland can provide regulating ecosystem services such as A) pollinator support through integrating flowering perennial wild plant such as elecampane (*Inula helenium* L.) in flower strips between fields, and B) erosion mitigation. Without proper soil cultivation and management, bare soil is prone to surface runoff after heavy rain events lowering the regulating ecosystem service of erosion mitigation, nutrient cycling, and carbon storage in soil.

A farmer can carry out the regulating services at farm level using a variety of ways. A high‐level summary of these services includes pollination control (**Figure** **3**A), erosion control (Figure 3B), genetic diversity control in the field, nutrition cycle control, and habitat control. There are also additional methods for providing these regulatory services, which will be discussed in greater detail below.^[^ ^35^ ^]^ Regulating services assist in the provisioning of resources, by providing additional services which aid in biomass creation. For example, by supporting genetic diversity across plant species for healthy diverse crops, farms can act toward agroecosystem resilience. Genetic diversity refers to genomic differences within populations of the same species and it helps species to adjust with rapidly changing environmental and/or climatic conditions.^[^ ^4^, ^47^ ^]^ Whereas phylogenetic diversity, refers to evolutionary distance among species^[^ ^48^ ^]^ which is hardly manageable at farm level. However, when we are applying agroecological theory the aim is to increase taxonomic (different species), phylogenetic (evolutionary variability) and genetic diversity (e.g., different varieties of rice). Furthermore, different crops can complement one another, resulting in synergy. Soil formation, water retention, and nutrient composition are all affected by ES.^[^ ^35^ ^]^ At system level, ES are critical as entire ecosystems rely on them for basic needs such as habitat development, biomass production, and atmospheric oxygen.^[^ ^49^ ^]^

#### 3.1.3 Habitat Services

**Figure 4:**
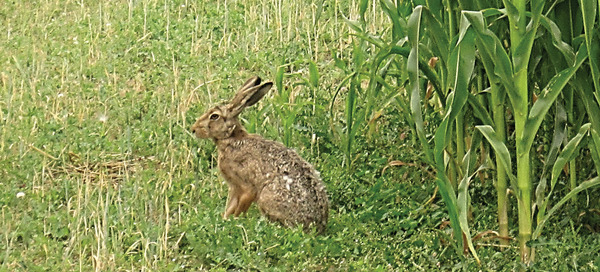
One of the small mammal species that can benefit from better habitat management in agriculture: The brown hare (*Lepus europaeus*, Pallas, 1778); here feeding along a field cultivated with maize (*Zea mays* L.). The brown hare relies on heterogeneous open field landscapes including fallow land and wildflower strips.^[^ ^50^, ^140^ ^]^

Habitat services concern the provision of habitat with food, nesting material, protection of various types of animals such as invertebrates, birds and small mammals^[^ ^50^, ^51^, ^52^ ^]^ (**Figure** **4**) from wind and weather (abiotic stressors), and from potential predators or rivals (biotic stressors).^[^ ^50^, ^53^, ^54^ ^]^ While there are no numbers yet available for grassland, the mean total monetary value of habitat services of cultivated land is about Int.$3016 per hectare and year, following De Groot et al. (2012).^[^ ^44^ ^]^

The habitat services of an agroecosystem can be more pronounced through landscape heterogeneity.^[^ ^55^, ^56^ ^]^ This can be done, for example, by increasing the density of contrasting landscape elements in an area such as arable land, hedgerows, grassland, and ponds as well as the respective transition zones.^[^ ^57^, ^58^ ^]^ Contrasting means here that the landscape elements differ strongly in their appearance, for example with regard to the types of flowers, the flowering durations, the height, quantity and type of above‐ and belowground biomass. The different appearances are accompanied by different living conditions for animals^[^ ^59^ ^]^ as well as abiotic effects such as wind reduction, microclimatic changes, improved nutrient recycling, etc. Hence, by increasing the density of landscape elements, more diverse living conditions for animals are created. In addition to the density, the arrangement of the landscape elements also plays an important role.^[^ ^58^ ^]^ It is essential to connect particularly ecologically valuable landscape element types with each other in the best possible way (habitat connectivity).^[^ ^60^ ^]^ However, in areas where the different landscape elements have been created over generations, it is difficult to optimize them. With regard to individual values of the farmers and values oriented toward the common good, a careful differentiation must be made in landscape planning interventions.^[^ ^61^ ^]^ But a better understanding of the influence of habitat services can help farmers to optimize the efficiency of small measures at farm level within the given scope of action. This is of importance for fauna and flora and can lead to higher agricultural biodiversity, and thus help to improve the resilience of an agroecosystem in the long term.^[^ ^62^ ^]^ Because landscape heterogeneity can only be managed at the farm level, it is so important for farmers, communities, landscape planners, and other decision makers to evaluate agricultural impacts on habitat services together.

#### 3.1.4 Cultural Services

Cultural services are possibly the least tangible because they can only be perceived and used subjectively.^[^ ^63^ ^]^ Their monetary value can therefore only be determined indirectly, for example via choice modeling or willingness to pay.^[^ ^64^, ^65^ ^]^ De Groot et al. (2021) estimated the mean total monetary value of cultural services provided by grassland and cropland to be Int.$1058 and Int.$1215 per hectare per year, respectively.^[^ ^44^ ^]^

**Figure 5:**
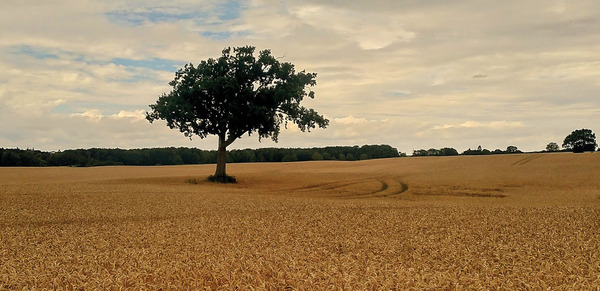
A lone tree in the middle of an intensively managed agricultural area in northern Germany (summer 2022). Even small landscape elements such as a single tree can contribute to the scenic beauty of the landscape, which merits consideration when evaluating cultural ecosystem services.

Among the highest forms of culturally valuable ecosystems would be primary vegetation (e.g., rainforests), which besides natural resources such as water, medicinal herbs, small wildlife and mushrooms also provides an inspiring abundance of colors, shapes, sounds and scents. Due to this broad ES portfolio, rainforests are now even considered a reason for so‐called urban–rural migration.^[^ ^66^ ^]^ But here, too, at least minimal anthropogenic development is needed to provide access at all, even if it is through a small trail.^[^ ^67^ ^]^ Hence, cultural ES include aspects like cultural identity, spiritual and symbolic interactions,^[^ ^23^, ^26^ ^]^ education, scenic beauty (**Figure** **5**), and recreational activities, and these services are only available to those who value them, and those who have access to them.^[^ ^67^ ^]^ As a result, while these services might not provide direct monetary advantages, they can be used to strengthen human–nature connection and motivate those who respect the life within or the overall appearance of ecosystems and want to preserve them.^[^ ^35^ ^]^ For instance, many farmers in southeast Asia worship crops, and celebrate sowing and harvesting.^[^ ^68^ ^]^

### 3.2 Synergies of Ecosystem Services—at Farm Level

In synergistic interaction, facilitating one of the ES will result in improving other ES and vice versa. Therefore, this section examines contributors to ES as well as potential synergistic interactions that may arise when employing more than one ES contributors. It is crucial to analyze ES that are provided at the farm level given that studies have shown that off‐field measures (e.g., hedgerows or vegetated field margins) can be significantly more effective in fostering biodiversity than in‐field measures only (e.g., organic management).^[^ ^55^, ^56^, ^57^, ^69^ ^]^ Thus, evaluating ES only at the field level could overlook key ES providers and supporters.^[^ ^70^ ^]^ In addition, the temporal component must be taken into account. This is because, managing ES in a stable manner can be difficult. Ecosystems can be understood as living organisms whose equilibrium is determined by intricate synergies and trade‐offs. To maintain an ecosystem in a healthy state, certain management strategies may be critical to promote a system balance.^[^ ^33^ ^]^ Agroecological farming approaches can help integrating necessary ecological steps into farm operations,^[^ ^12^ ^]^ aiming to preserve the natural order of the agroecosystem.^[^ ^71^ ^]^ The following section will provide an overview of key‐ES and ‐agroecological strategies^[^ ^34^ ^]^ such as insect pollination and biological control, cover cropping, and intercropping used on the farm, highlighting their synergies and trade‐offs. Since insect pollination and biological pest control are among the most relevant ES,^[^ ^53^, ^72^ ^]^ we consider them as the first examples.

#### 3.2.1 Insect Pollination and Biological Pest Control

Insect pollination is essential to the integrity of any natural ecosystem.^[^ ^72^, ^73^, ^74^ ^]^ In fact, to reproduce and generate yield, over 75 percent of all food crop species rely on insect pollination. Bees, wasps, flies, beetles, butterflies, moths, and hummingbirds are some of the most abundant pollinators.^[^ ^35^, ^75^ ^]^ Good habitat management is the first step in controlling insect pollination at the farm, and to enhance the quantity of pollinators, one should learn about their natural habitat.

For example, honeybees are a major contributor to the provisioning of many foods including fruits and vegetables, and require a pollen‐rich habitat. Areas with broadacre row crop fields, such as maize (*Zea mays* L.), soybean (*Glycine max* L. Merr.), rice (*Oryza sativa* L.), cotton (*Gossypium* L.), which are wind‐pollinated and/or mass‐flowering crops,^[^ ^76^ ^]^ are considered the least suitable for attracting and supporting pollinators such as honeybees. To provide good beehive habitat, food and water sources, as well as protection from natural predators (farm level) must be considered.^[^ ^77^ ^]^

Furthermore, it is vital to decrease or eliminate the use of chemical–synthetic plant protection measures (CSPM) such as insecticides and herbicides at the farm^[^ ^78^ ^]^ by introducing biological agents—also known as biocontrol agents or small predators.^[^ ^79^ ^]^ CSPM have proved to be hazardous to various pollinators.^[^ ^75^, ^78^ ^]^ Thus, reducing or even avoiding the use of CSPM can result in increasing pollinator populations at farm level^[^ ^78^ ^]^ and thus creating synergies between ES pollination, biocontrol, and nursery service.

Another method for increasing pollinator presence is allowing weeds (generally wild flowering plants) to remain at the field's edge. Weeds can provide additional food for pollinators while also protecting them from natural predators.^[^ ^56^, ^80^, ^81^ ^]^ Pollinators such as the honeybee and wild bees require suitable nesting sites and the right type of flowers (nectar and pollen) at the right time.^[^ ^80^, ^82^ ^]^ In rather homogeneous agricultural landscapes, there are usually long periods when no or only few plants are in bloom and thus feed is scarce for pollinators. These gaps in feed availability are particularly critical for wild bee species in late summer and autumn, because it is then that they depend on special nectar and pollen from wild flowers.^[^ ^80^ ^]^ Therefore, surrounding landscape elements containing the required structures and nesting materials such as trees and shrubs should not be cleared, as they may be essential for nesting and overwintering of pollinators and biocontrol agents.^[^ ^55^, ^57^ ^]^ This in turn, provides biological pest management solutions,^[^ ^33^, ^78^, ^79^, ^83^ ^]^ with the use of naturally occurring or commercially available biocontrol agents such as *Trichogramma ostriniae* against corn borer (*Ostriniae nubilalis*) being considered ecologically sound.^[^ ^78^, ^84^, ^85^ ^]^ In addition, farmers can improve and stabilize yields and quality of their agricultural products, control weeds and reduce biodiversity losses by using organic inputs and biological agents instead of hazardous fertilizers and pesticides.^[^ ^71^, ^78^, ^79^ ^]^ Additionally, private gardens in close proximity to agricultural managed fields can play a crucial role in providing nutrition but also nesting sites and feed for pollinators.^[^ ^86^ ^]^ The promotion and active use of biocontrol agents is therefore seen as contributing to the long‐term resilience of agroecosystems.^[^ ^78^, ^87^ ^]^

#### 3.2.2 Cover Cropping

The abovementioned strategies to reduce CSPM use^[^ ^78^ ^]^ can benefit from a mixed approach that includes cover crops (also known as catch crops).

Farmers worldwide are embracing conservation practices such as cover cropping combined with no‐tillage approach to produce revenue.^[^ ^88^, ^89^, ^90^ ^]^ Cover cropping occurs in between harvests, to provide additional ES to the field such as reducing both nutrient leaching and erosion,^[^ ^91^, ^92^ ^]^ and to increase the quantity of in‐field crop residues which improves soil processes. Therefore, the purpose of a cover crop is often to increase soil fertility, via positive change in chemical, biological, or physical properties.^[^ ^93^ ^]^ Thus, cover crops are usually sown following harvest and extinguished prior to planting of the following main crop.^[^ ^91^ ^]^ Furthermore, a cover crop can also be grown as living mulch together with a main crop,^[^ ^94^, ^95^ ^]^ but this could also be considered as intercropping. Either way, there is a wealth of knowledge about the potential positive effects of cover crops on pollinators and biocontrol agents.

For example, cover cropping research at the University of California, USA, has demonstrated the increased presence of parasitic wasps, and biocontrol agents in mixed vineyard systems which attract pollinators as well as predatory arthropods. This combined strategy lowered the number of pests, such as the vine mealybug, while significantly increasing the number of pollinators.^[^ ^96^ ^]^ The vine mealybug is harmful to the crop, and if not controlled, will destroy the vineyard. The Agricultural Commissions Office, in Napa Valley, California is currently running a county wide program to control the spread of mealybug. The program involves introducing parasitic wasps such as *Anagyrus* species to the vineyard as the natural predator of the mealybug.^[^ ^97^ ^]^ Even though parasitic wasps are efficient at managing pests including mealybug, they rely on other food sources, such as nectar, which is only available during crop's flowering. In the absence of flowering cash crops, there are other farming strategies which can support *Anagyrus* and other biocontrol agents including cover cropping and intercropping.

**Figure 6:**
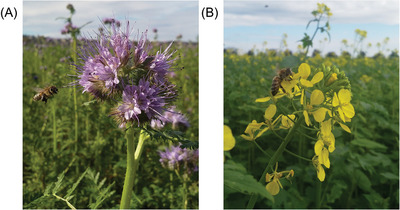
Impressions of cover crops A) purple tansy (*Phacelia tanacetifolia* Benth.), and B) white mustard (*Sinapis alba* L.) at full blossom during autumn. Photos taken in Filderstadt, Germany, on 16th October 2022.

Various cover crops such as white mustard (*Sinapis alba* L.) and purple tansy (*Phacelia tanacetifolia* Benth.) (**Figure** **6**) are known to can act as natural buffer during nonflowering stages.^[^ ^98^ ^]^ One of the many other large groups of biocontrol agents benefiting from this agricultural practice besides *Anagyrus* are ground beetles. Many of those beetles belonging to the ground beetles are natural predators of field pests such as caterpillars, slugs, and grasshoppers. During autumn and winter, ground beetles require natural buffer zones of cover crops that provide a sustainable feeding source. Such natural buffers can also be maintained by leaving some fields grassy or by building a beetle bank.^[^ ^86^, ^99^ ^]^

**Figure 7:**
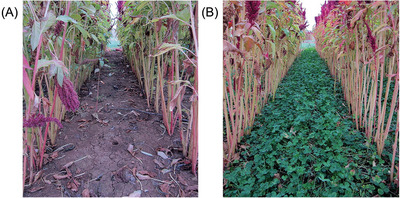
Amaranth (*Amaranthus hypochondriacus* L.) grown for biogas production in southwest Germany A) without, and B) with low‐growing white clover (*Trifolium repens* L.) as living mulch.

The appropriate selection of a cover crop and its implementation in a farming system should be based on a set farming objective. In soil, cover crops can be used to increase the organic content, reduce weeds, capture water, control erosion, and manage nitrogen, along with other plant nutrients.^[^ ^91^ ^]^ Cover crops can be sown after the main crop as well as simultaneous, for example low‐growing white clover under maize or amaranth (*Amaranthus hypochondriacus* L.) (**Figure** **7**).^[^ ^100^ ^]^ Low‐growing white clover then serves as a so‐called living mulch, which has already begun to cover the soil between the planting rows by the time the main crop is harvested.^[^ ^100^, ^101^ ^]^ In this way, the additional ES already mentioned can be provided even better than if the cover crop had to start developing from the time of sowing shortly after the main crop harvest.

However, cover crops can also be sown as mixtures to harness further ES benefits. Mixtures of cover crops (cover‐crop intercropping) can fulfill numerous goals through multifunctionality while building synergy between species.^[^ ^102^ ^]^ For example, a legume species grown with grain, radish or other nonlegume species makes for good cover crop pairing,^[^ ^103^, ^104^, ^105^, ^106^ ^]^ because the combination provides for greater production. Legumes are a source of biological nitrogen fertilizer for other crops by fixing atmospheric nitrogen via rhizobacteria,^[^ ^106^ ^]^ are less dependent on soil nitrogen and release nitrogen when decaying which can build soil organic matter and supply other crops. Grain, radish, or other nonlegumes, on the other hand, rely on soil nitrogen but vary in their nitrogen demand. Scavenger crops including grain and radishes thrive in the presence of nitrogen‐fixing legumes, and therefore complement one another.^[^ ^107^ ^]^ Under best management practices, this can lead to increased land equivalent ratio compared with sole cultivation of the crops.^[^ ^108^ ^]^ Improved soil root structure aiding erosion control is another advantage of scavenger plants. Through the left‐over crop residues, cover crops add organic material to the soil which recycles nutrients, builds soil organic matter and provides plant available mineral nitrogen (and other nutrients) as organic compounds (such as amino acids) and after microbial mineralization in the following growing season.^[^ ^109^ ^]^

#### 3.2.3 Intercropping

Intercropping or mixed cropping techniques can be successful strategies for increasing agrobiodiversity at farm level.^[^ ^83^, ^110^ ^]^ Intercropping entails planting different crops simultaneously^[^ ^111^ ^]^ in order to achieve beneficial interaction or synergies between two or more crops. Therefore, intercropping followed by cover crops can further deepen ecological values throughout the year with low risk of compromising ecosystem integrity by further providing nursery services for animals during winter and contributing to biocontrol.^[^ ^112^, ^113^ ^]^

**Figure 8:**
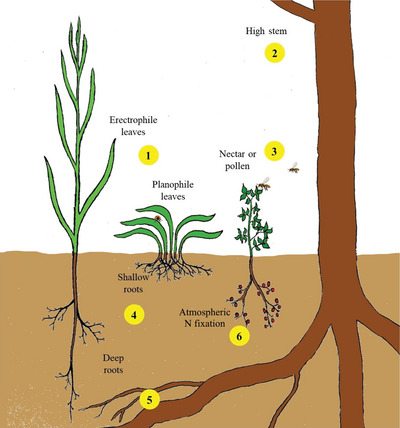
Schematic impression of some basic crop characteristics in intercropping systems. By combining plants with different leaf angle distributions erectophile and planophile 1), the light use efficiency of the cropping system can be improved. Tall woody crops 2) help reduce erosion after extreme events and light stress on hot days or during dry periods. Flowering crops 3) provide feed for numerous insects which helps improving and stabilizing numerous food chains within the agroecosystem. Combinations of shallow and deep rooting crops 4) enables more efficient use of soil water and nutrients compared with mono‐cropping systems. Dead wood in the soil 5) increases activity of soil mycorrhizal fungi improving the conditions for plant–fungi symbioses. Integration of legume species 6) to intercropping systems allows for additional input of atmospheric nitrogen (N) to the system feeding other crops after being mineralized again.

In agricultural systems aimed at maintaining the integrity of soils, ecosystems, and the environment, intercropping is a common practice^[^ ^114^ ^]^ allowing for a higher land equivalent ratio,^[^ ^108^ ^]^ as was also mentioned above with regard to cover cropping. With intercropping it is possible to achieve ecological intensification while maintaining the integrity of the ecosystem.^[^ ^111^ ^]^ When considering crop biomass production and yield, Liebig's rule of the minimum^[^ ^115^ ^]^ applies also to intercropping or cover cropping and is an important concept that stipulates that a crop's productivity limitation can be traced to one critical component. This limiting factor is the availability of water, sunlight, or one of the 14 essential (and additional beneficial) crop elements, also termed nutrients.^[^ ^111^ ^]^ Crops and their interactions with the environment, whether above or below ground (**Figure** **8**), can often compensate for limiting factors^[^ ^102^, ^111^, ^114^ ^]^ or reduce abiotic stress factors.^[^ ^19^ ^]^ In agroforestry for instance, trees can improve growth conditions for annual or winter‐annual food crops via reducing heat stress in summer, potentially contributing to mitigate climate change impacts on crops by creating micro‐climate effects. Further advantages include weed control, pest regulation, for example, by using the push‐pull approach, which helps reduce a pest infestation of a target plant (e.g., maize) by an intercropping plant (e.g., *Desmodium adscendens*) that emits a chemical repellent (the pest is driven away from the target plant by the repellent).^[^ ^116^, ^117^ ^]^ Thus, intercropping has benefits in addition to facilitation, sharing of resources, and complementary interactions,^[^ ^111^ ^]^ as shown in Figure 8. Key advantages include plant macro‐ and micronutrient acquisition and, in the case of nitrogen biological fixation, fungal interactions, and supporting canopy and root architecture. Consequently, intercropping systems can also benefit from engineering or breeding of plants for desired physiological traits such as improved nutrient uptake efficiency or morphological traits such as different leaf angle distribution (planophile/erectrophile), and rooting depth.

Overall, diversifying farm systems with genetically and functionally diverse crops are most likely to support and contribute to a range of ES. And it is well known that perennial crops such as *Miscanthus* ANDERSSON enable total ES at two to three times the pure biomass‐linked monetary values, and help to equalize peak workloads of the farm.^[^ ^118^, ^119^ ^]^ Therefore, strip‐intercropping approaches (stripwise combinations of annual and perennial crops) can provide very special financial incentives for farmers at the farm level, paving the way for greater scope to develop and implement more sustainable (e.g., more biodiversity‐friendly) farm designs.^[^ ^120^, ^121^ ^]^ However, there are several burdens to be overcome such as the technical extension service to provide guidance to farmers, the additional labor effort required and the need to adapt agricultural machinery to the layout of intercropped farms. And a key economic issue would be who could pay for the additional ES, the government, or other stakeholders besides the farmer? This question will be addressed later in this study.

When selecting the appropriate combination of plant species for cover or intercropping, it is central to design communities in a way to deliver a suite of ES at the farm level. A good starting point is to identify and support the limiting factors on the farmland or in the region in which the farm/s operate. Thereafter, databases are required to compile knowledge on ES‐relevant plant traits and identify preferably native species and species mixture characteristics considering both tradeoffs and synergies between the crops and their environment^[^ ^122^ ^]^ toward optimizing the overall ES performance.^[^ ^111^ ^]^ For instance, these databases can help farmers who are looking for existing plants with beneficial characteristics at farm level, or explore which new additions to the farm could be beneficial.^[^ ^123^ ^]^ However, the task of selecting and combining plant species should be done by experts in collaboration with farmers, as the introduction of a new species could have significant consequences to the fauna and flora of the field^[^ ^35^ ^]^ and, in some cases, of the region.^[^ ^124^ ^]^ Faucon et al. (2017) have shown what a challenging task such an initial research and database setup would entail, but also that a large body of knowledge already exists about individual components of such a database.^[^ ^122^ ^]^

In terms of fungal relationships, the sharing of resources such as nutrients and minerals is typically achieved through the use of fungal networks.^[^ ^111^ ^]^ Most of the world's plant species in land‐based biomes are known to create symbiotic interactions with fungal networks.^[^ ^125^ ^]^ However, nutrient sharing can also take place as a result of recycled nutrients given above ground by plant senescence (leaf litter)^[^ ^126^ ^]^ or underground by the decaying root system (root turnover).^[^ ^111^ ^]^

With regard to plant architecture or spatial separation, the combination of plants and their niche separation can enhance complementary utilization of resources such as light, water, and nutrients.^[^ ^19^ ^]^ The spatial and temporal interactions of diverse species living in heterogeneous settings are characterized by niche separation. Niche separation is an ecological concept that explains how certain organisms may grow over their theoretical capacity while resisting the competitive exclusion principle.^[^ ^127^ ^]^ A spatial niche is one that benefits from varying plant distributions and heights (Figure 8). It can, for example, enable optimal sunlight absorption of species that live in proximity. It may also have different root structures or lengths, which may help plants improve nutrient absorption by spreading nutrients over the soil surface (Figure 8).^[^ ^111^ ^]^ A temporal niche is one that provides synergistic interaction between two species with differing growth trajectories. For instance, pairing a fast‐growing species with a short life span with a slow‐growing long‐lived one results in a temporal niche for both,^[^ ^127^ ^]^ avoiding competition and resultant growth limitations and improving diversity.

A key message is that a single limiting factor can often be addressed to overcome bottlenecks affecting the overall yield, for example by adding water or fertilizer. However, to maximize the output of all ES, one should consider balancing the net benefit of all services. In doing so, one takes into account the entire system's balance, including pests, pollinators, nutrients, soil, water, and sunlight.^[^ ^111^ ^]^ In addition, the cost at which an ES is recycled or marketed should be analyzed, as was shown with the example of *Miscanthus*.^[^ ^119^ ^]^

### 3.3 Trade‐Offs of Ecosystem Services—at the Farm Level

Over the past 50 years there has been a trend of rapid degradation of ES.^[^ ^73^ ^]^ In agriculture, the concentrated use of provisioning services is viewed as a driver of the degradation of other ES, with regulating services being the most damaged. Agricultural land use dominates all other land use types; with almost 5 billion ha worldwide, crop, pasture, and range areas accounted for 38 percent of global land surface area,^[^ ^128^ ^]^ and it has undergone significant intensification in recent years. In this agricultural intensification lies the most typical sort of trade‐off in agriculture: the trade‐off between providing natural resources (i.e., biomass) and sustaining other regulatory or cultural functions as mentioned above.^[^ ^30^ ^]^ Going too far in one way, such as boosting the provision of a single item (e.g., a food or fodder crop), can result in lower resilience of the agroecosystem,^[^ ^47^ ^]^ nutrient depletion and other contaminants produced by significant run off.^[^ ^129^, ^130^ ^]^ However, to sustain human existence, agriculture must meet the growing need for biomass to provide food, feed, biobased products and bioenergy, thus striking a balance can be difficult.^[^ ^35^, ^131^, ^132^, ^133^ ^]^ In Europe, the integration of Agri‐Environmental Schemes (AESs) reserves areas, so to speak, for numerous ES except those directly serving the provision of food crops or renewable resources while the farmers are enabled to compensate for the loss of income through appropriate subsidies^[^ ^134^ ^]^ for which more than €22 billion were spent by the EU between 2007 and 2013. According to an extensive recent review of Paulus et al. (2022), low yielding sites are mostly used for this purpose.^[^ ^134^ ^]^ This represents a classic land use conflict due to a tradeoff between providing and regulating ESs: low‐yield sites, most often referred to as marginal agricultural land, are considered as potential cropland for nonedible (industrial) crops to avoid land‐use conflicts with food crop production.^[^ ^135^, ^136^, ^137^, ^138^, ^139^ ^]^ There are approaches to design a use of marginal land with industrial crops in a way that nevertheless also performs regulating functions along the lines of AESs,^[^ ^19^ ^]^ such as perennial biomass crops,^[^ ^19^, ^136^, ^137^ ^]^ flowering region‐native crops,^[^ ^140^, ^141^ ^]^ or increasing the landscape heterogeneity.^[^ ^142^ ^]^ Nevertheless, the question remains to be critically discussed and considered on a case‐by‐case basis whether land sharing (industrial crops as AESs) or land sparing (either industrial crops or AESs) is preferable, also, for example, under aspects of air quality regulation (carbon sequestration in the soil) and environmental impacts such as acidification, eutrophication, and resource use.^[^ ^143^, ^144^ ^]^

Another example for this dilemma, the trade‐off between provisioning and regulating ESs, are extensive cropping systems such as organic farming.^[^ ^20^, ^78^, ^145^ ^]^ Organic farming can be seen as a kind of land sharing approach, as it allows for, to a certain extent, restored ecosystems at farm level^[^ ^3^ ^]^ which support important ES other than provisioning ESs. However, the use of chemical–synthetical plant protection measures and mineral fertilizers is prohibited in organic farming, which places great demands on the production of sufficient quantities and qualities of food and fodder crops.^[^ ^78^ ^]^ Even though there are numerous promising strategies for this from agroecology (traditional agricultural knowledge),^[^ ^12^, ^146^ ^]^ organic farming mostly achieves significantly lower yields in comparison to conventional agriculture.^[^ ^147^, ^148^ ^]^ As a result, extensive cropping systems often require more land to produce the same amount of food.^[^ ^78^ ^]^ This also heats the land sharing/sparing debate, i.e., whether it makes more sense from a biodiversity perspective to use the current agricultural land intensively and spare the rest of the land for semi‐naturals habitats instead of using all available land extensively.^[^ ^149^, ^150^ ^]^ However, the use of diversification practices such as intercropping can allow for a significant reduction of the organic to conventional yield gap while simultaneously fostering biodiversity thereby offering the opportunity of creating a win–win situation.^[^ ^33^, ^57^, ^151^ ^]^

In addition, cultural services are also often regarded as resource provisioning trade‐off, because intense provisioning frequently leads to the loss of recreational value at farm level.^[^ ^63^ ^]^ Although the recreational value may seem marginal given the drastic impact of biodiversity losses on the resilience of agroecosystems, its multiplier effect should be carefully considered. In this regard, it is believed that more tourism‐friendly landscapes that can be well used for local recreational purposes^[^ ^63^, ^152^, ^153^ ^]^ could help raise awareness among the population for more sustainable consumer behavior. This would reduce the pressure on farmers to grow more and more at lower and lower prices (sole focus on efficiency and productivity).

Thus, any actions aiming at lowering or correcting the adverse trade‐offs associated with intensive provisioning must be directed toward the agriculture sector.^[^ ^154^ ^]^ Unfortunately, when it comes to ES management decisions, the provisioning ES comes first, followed by regulating, habitat, and cultural services. This causes ecosystems to become unbalanced since one service is emphasized over others.

Geographical, temporal, and reversible aspects should be considered when weighing in on ES trade‐offs.^[^ ^2^ ^]^ For instance, in hydrological situations where water must be re‐distributed, spatial dimensionality is critical (i.e., to irrigated crop land). With regards to land management, most decisions include a time‐sensitive trade‐off, such as whether to degrade fertile soil and groundwater now or later. In the event of an unanticipated outcome, the amount of reversibility, or how quickly the trade‐off may be reversed, must also be considered.^[^ ^2^ ^]^

The bundling of ES is a suitable way of analyzing their trade‐offs. According to studies, ES are frequently found in pairs at different temporal and geographical domains.^[^ ^155^ ^]^ Moreover, it has been discovered that the negative or beneficial effects of ES may be matched with current bundles.^[^ ^156^ ^]^ As a result, there is a need for ES research to develop innovative ES bundles, which would enable even more efficiency in examining synergies and trade‐offs between the services.^[^ ^157^ ^]^

### 3.4 Stakeholder Analysis

**Figure 9:**
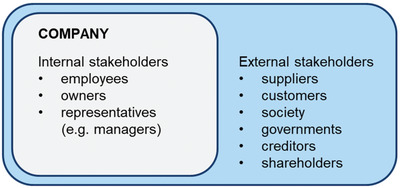
Example of an internal and external stakeholder model (based on ref. [159]).

The widely used term stakeholder analysis defines a broad set of tasks related to identifying or classifying key stakeholders involved in a process or the business of a company (**Figure** **9**). Consequently, there are different models and frameworks for conducting the analysis, thus one must come up with a best fit scenario given the situation. It is said that a fundamental component of ES research is the involvement and co‐creation of research projects with relevant stakeholders. It may be difficult to incentivize stakeholders to foster ES without economic reasons—despite the fact that there are different worldviews in the context of ES among farmers worldwide.^[^ ^26^ ^]^ However, educating them about the importance of different ES may encourage them to incorporate ecological practices into their agricultural operations. When it comes to ES research, scientists are usually met by policy change restrictions. In truth, stakeholder participation determines policy shifts more so, than the findings on their own.^[^ ^158^ ^]^

For a stakeholder analysis, it is therefore common to start with a broad scope, via classifying primary groups of stakeholders, and then subdividing them into specific subcategories. Based on an example using the Responsible Research and Innovation (RRI) framework (adapted from^[^ ^159^ ^]^) depicted in Figure 9, the primary classes are defined as Internal and External stakeholders. Internal stakeholders are then further categorized into workers, managers, and holders (i.e., of the farm). For the external stakeholders they are divided into suppliers (i.e., of inputs or services), customers, society, government, creditors, and shareholders.^[^ ^159^ ^]^

The RRI model is derived from conventional stakeholder theory, however it differs by incorporating diverse stakeholders (i.e., NGO's, Researchers, Unions, etc.). The RRI model is founded on principles of corporate responsibility, and thus takes into consideration other factors besides profit, such as social impact. In terms of addressing ES, and its abstract nature (i.e., cultural services), this model is better suited than a traditional profit‐based model, as it allows for greater flexibility in assessing stakeholders and their interests. The RRI framework also accounts for direct and indirect incentives, which relates to the benefits represented in the cascade model below (Figure 9).^[^ ^159^ ^]^ This is relevant to ES, as society usually tends to choose direct benefits over indirect benefits. This phenomenon explains why society favors certain options that offer an inherent benefit, over ones where the benefits develop over time. Thus, direct, and indirect incentives are likewise applicable, as they are indicative to societies preferences.^[^ ^160^ ^]^ The third part to the RRI framework consists of classifying incentives as instrumental or noninstrumental. Instrumental incentives are those that incentivize a call to action. Whereas noninstrumental incentives are those which aren't related to any agenda. An example of an instrumental incentive is one that awards positive behavior (i.e., reducing carbon emissions). Examples of noninstrumental incentives are profit, or a company's continuity. In summary, the RRI model is one based on impact and includes a wide variety of stakeholders. The framework tries to address this model using three layers: 1) internal versus external stakeholders, 2) direct versus indirect management activities and benefits, and 3) instrumental versus noninstrumental incentives.

**Figure 10:**
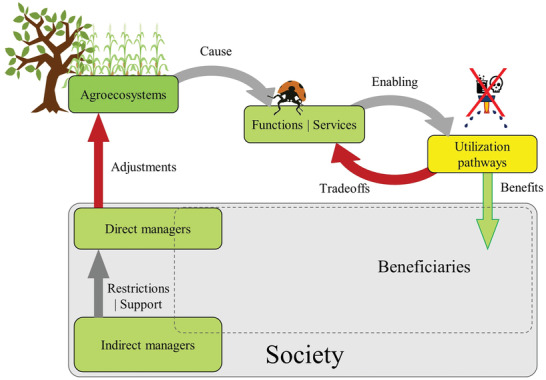
Schematic depiction of the ecosystem services cascade framework (based on ref. [161]).

A factor that is relevant to ES, is the distributed roles of power distribution, power relations, the evaluation process itself and the governance over ES.^[^ ^26^ ^]^ An empirical study attempts to address this with a cascade model.^[^ ^161^ ^]^ The cascade model shows the ES cascade, and how it is influenced by stakeholders. Which is dependent on direct or indirect management decisions along the cascade. This type of analysis uses direct and indirect management activities, as its primary framework. The framework is then further subdivided, by the three sets of ES along the cascade. **Figure** **10** depicts the cascade model, along with direct and indirect management roles.

The cascade framework (Figure 10) starts at the ecosystem‐level were activities attributed to the alteration of the ecosystem itself are considered. The ensuing stage deems ES at the service level, here activities influencing ES are considered (i.e., provisioning, supporting, regulating, and cultural services). At last, the use‐level associates benefit that humans can consume from the ES. Figure 10 shows the cascade model where all levels of the cascade are influenced by direct and indirect management. Although all stages in the cascade can be shaped by management, only the benefits derived from the use level are consumable. This is meaningful in the sense that society prefers immediate, tangible, consumable benefits over gradual, abstract ones.^[^ ^161^ ^]^ This distinction between tangible and abstract defines how society prioritizes inputs that provide an immediate advantage over benefits that arise across time and space. This notion explains why people are less concerned about unfavorable events that occur in the distant future.^[^ ^160^ ^]^ Figure 10 takes this concept into account, as only the final step along the cascade depicts the benefits from ES allocated to society. Figure 10 also depicts how indirect managers, and society can influence direct management by either restricting or aiding its activities.

**Figure 11:**
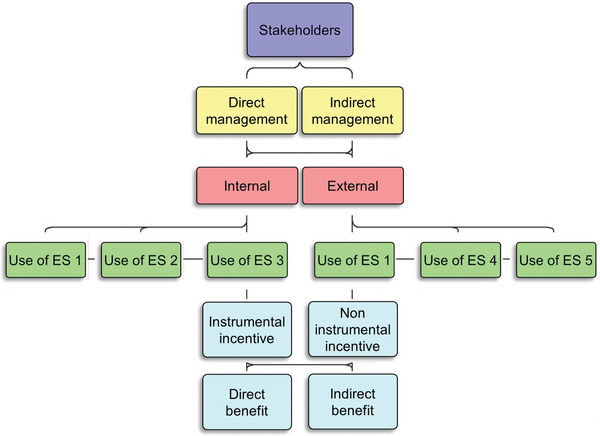
All stakeholders, and their direct and indirect management roles in ecosystem services (ES) cascade (created with the ArisExpress software tools and derived from the proposed technique in Figure 9, as well as the framework in Figure 10). ES1‐5 represent schematic ES.

**Figure** **11** shows a visualization representing a method which unites both the RRI, and ES cascade framework. This method is intended to show how various management activities affect ES. Because this strategy is activity‐centric, its goal is to emphasize managerial activities that have an influence on ES benefits. By assigning both frameworks in the manner discussed below, one may create a link between the activities that influence ES and the intended ES advantages. Developing a link between the two might help policymakers and stakeholders make better ES decisions.

The layout in Figure 11 conceptually starts with all stakeholders, and their direct and indirect management roles. The subsequent red section represents internal and external activities. It may make semantic sense to invert the yellow and red rectangles, as stakeholders could be classified from the start as internal or external. However, conceptually, the activity itself determines if the stakeholder is acting internally or externally. Therefore, the activity must come first. Understanding how direct and indirect activities occur internally and externally helps clarify this notion. Here by example; a farm worker, who is considered an internal stakeholder (based on the RRI process), can directly influence ES at the field, or indirectly as part of an organization (i.e., farm bureau).^[^ ^159^, ^161^ ^]^ Vice versa, a society, considered an external stakeholder, can directly manage and influence ES. Directly, when a small‐scale community constructs a water reservoir to assist with farm irrigation. Or indirectly, when a community declares a natural area, as a protected sanctuary.^[^ ^159^, ^161^ ^]^ In the case of the community, a widespread drought affecting the community, would change its stake, which is now internalized. In the case of the worker, the stake becomes externalized when acting through the institution. Thus, one can see how the activity affects the internal and external stake. In theory, stakeholders of an entity can be both internal and external to the organization, which solidifies the notion.^[^ ^162^ ^]^ Following stakeholder attributes, the management activity is assigned to the appropriate level in the ES cascade. This stage is essential as it demonstrates where the ES comes from and can be used to explain which activities within the cascade are influencing the final product, the ES benefits. In this case, the cascade related attribute is separate, and does not determine any prior attribute. The cascade attribute is used to signify the responsible stage for the ES benefit. The following section (in blue) represents the incentives, grouped together (Figure 11). This is done as there is no particular order required in assigning the attributes. However, they appear subsequent to the cascade attribute, as the incentive or benefit can only stem from the use‐level. These final attributes could provide information to policy makers, in terms of whether a specific benefit was effectively incentivized, via instrumental or noninstrumental means. In addition, policy makers can better understand the distinction between direct and indirect benefits, to know which benefits are undervalued by society. For instance, policy makers could assess the analysis and conclude that few management activities are addressing indirect benefits, such as groundwater management. Although groundwater management is important to a society, in this example it is overlooked. Thus, policy makers can conclude that more incentives for groundwater management are needed.

Additional criteria related to internal stakeholders such as workers, managers, and holders, or external criteria such as suppliers, customers, society, government, creditors, and shareholders are not depicted. However, these criteria could be assigned independently at the stakeholder level, but not subsequently, as in this case, it is not coupled with the internal or external attribute. For example, one could do a preliminary evaluation by implementing the criteria at the start to determine all key stakeholders involved in the process. In any case, more data collected in the fields will be needed to better evaluate farm‐level relationships and interactions. This is where digital farming may be relevant for the future.

### 3.5 Outlook—The Role of Digital Farming for Ecosystem Service Assessment

**Figure 12:**
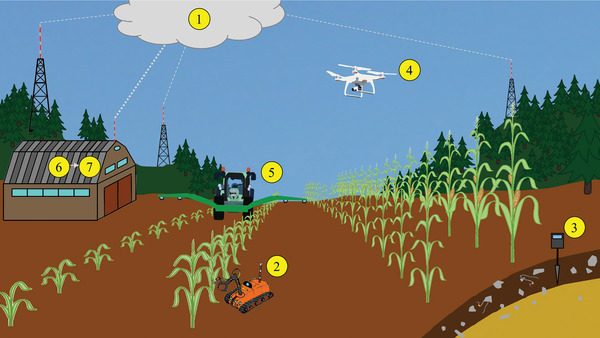
Overview of proposed additional functions in modern agricultural systems to support ecosystem service (ES) measurement and assessment during actual technology application: 1) weather data cloud, 2) field robots collect additional biodiversity data during the growing season, 3) soil sensors and weather data loggers help monitor above‐ and below‐ground microclimates, 4) drones collect additional plant stand data during the growing season, 5) tractors collect additional plant stand data, e.g., in sensor‐driven online measurements of nitrate content in leaves, 6) the farm office compiles data, and 7) artificial intelligence‐driven ES assessment provides the farmer with important information on ES performance both over time (growing season, full year, etc.) and considering single ES or multiple sets of ES (bundles).

Digital farming technology provides various services to agricultural production activities. Technology could however be adapted with additional features to support the measuring and valuation of ES during the actual application of technology (**Figure** **12**). The goal is to identify dimensions in terms of area, location, size of fields amongst others, which are relevant to understand interdependencies, causalities and provide the basis for estimations on the value of biodiversity in particular in identifying bundles of ES as explained earlier. This may bear the potential to increase both the use of digital farming technology and help find a way to monetarize ES, which is a challenge. Such an approach is based on the large body of research done in modeling of farming systems and decision support systems (e.g., to understand climatic influence on crop farming).^[^ ^163^, ^164^, ^165^ ^]^ This suggestion does, however, not come without its challenges.

The idea is that many digital tools already use data sets for calibrating/training underlying models or even during application which entail information also relevant for the identification and valuation of ES such as plant varieties, critical soil moisture levels etc. Those technical possibilities are currently not specifically explored for the purpose we suggest but for the agricultural production activities such as mechanical weed control or measuring the growth development of a crop for estimating the application use of chemicals and finally to provide decision support to the farmers.^[^ ^166^ ^]^ Technically, attaching an additional feature, such as an additional camera or a hard drive to store data for the analysis, i.e., for example to document the varieties of weeds in the field or document 3D dimensions of a location is feasible and not a technical challenge.

On the technological side, more data and information collection rather brings ethical dimensions to the table such as property rights, and the question who should process the data but also data protection regulations are to be considered. In a next step, estimating the value of ES extends to the questions which technology may be the best to assess what kind of indicator, maybe even depending on whether it is a biotic or nonbiotic.^[^ ^167^ ^]^ Elmiger et al. (2023) mentioned apps to determine plant species and argue that satellites might not be very well suited for assessment, they refer mainly to ES in grassland landscapes.^[^ ^167^ ^]^

The idea of this study goes beyond this and tries to envision possibilities particularly in technologies applied in crop production and measurements in between applications. Sensors attached to machines could measure attributes contributing to ES such as air temperature and moisture between fields, when driving from one field to the other (Figure 12). Indication for landscape heterogeneity, phytosanitary conditions, etc., could be obtained by using robotic technology to actually identify the weeds during weed control. Improved knowledge on the abundance and diversity of weed populations helps estimating the monetary value of several ES such as genetic resources (a provisioning service), pollination (a regulating services) and nursery services (a habitat services) (Figure 1). Further examples could be the measurement of emission when applying liquid manure through sensors attached right to the application technology. The goal of the measurements should be, amongst others to “*disentangling* *nature's contribution in complex agri‐environmental systems*”^[^ ^168^ ^]^ from other contributions, such as management or infrastructure. This is important to design the right policies for example.

On the data management side, methods used in the industry to anonymize data for various reasons could be used in the form of generating synthetic data^[^ ^169^ ^]^ to only extract the relevant information without risking to lose the quality of data but providing data protection to the ones who are willing to contribute to the analysis. This positive rebound effect could help to identify a strategy to compensate farmers for their ES contributions but also help communicate about the ES and increase visibility of actual state.

The application of adaptive agroecological practices promises to ensure sustainable and high‐quality crop productivity and improve soil carbon pools, nutrient balance, and soil biodiversity.^[^ ^10^, ^33^, ^34^, ^78^, ^116^, ^146^ ^]^ Such an approach is key to restoring a bundle of ES without compromising farm integrity, thus contributing to the advancement of combined land sharing and sparing solutions.

## 4 Conclusions

This study reviews how different ES management activities at farm level can affect key ES along the ES cascades. For instance, if one was to assess each activity for its impact, one could evaluate whether more ES are generated internally or externally. Furthermore, this method might be used to identify if the internal or external stakeholders share equal control over the ES at farm level. It can also assist with better ES management and policy decisions along the ES cascades. Additionally, the potential benefits of adapted digital farming approaches combined with artificial intelligence‐driven ES assessment on better ES management at farm‐level are discussed. Altogether, this can help to decisively improve the social‐ecological sustainability and resilience of agricultural production in the long term.

## Conflict of Interest

The authors declare no conflict of interest.
